# Alexithymia, social support, depression, and burnout among emergency nurses in China: a structural equation model analysis

**DOI:** 10.1186/s12912-021-00702-3

**Published:** 2021-10-10

**Authors:** Juhong Pei, Xinglei Wang, Haixia Chen, Hongchen Zhang, Ruiling Nan, Jing Zhang, Xinman Dou

**Affiliations:** 1 The first clinical medical college, Lanzhou University, Lanzhou, Gansu China; 2 School of Nursing, Lanzhou university, Lanzhou, China; 3 Department of Nursing, Lanzhou University Second Hospital, No. 82, cuiyingmen, Lanzhou, 730000 Gansu China; 4 Department of EICU, Lanzhou University Second Hospital, Lanzhou, Gansu China

**Keywords:** Alexithymia, Burnout, Social support, Depression, Nurses, Structural equation modeling, China

## Abstract

**Background:**

Several factors are associated with the incidence of burnout, including alexithymia, social support, and depression. The relative importance of these three key parameters as mediators of burnout, however, is not well understood. In addition, there have been few studies to date specifically examining the association between alexithymia and burnout among nurses in China.

**Purpose:**

To evaluate the relationship of burnout with alexithymia, social support, and depression across emergency department nurses in China.

**Methods:**

This descriptive, cross-sectional survey was conducted using a convenience sampling methodology to survey nurses responsible for direct emergency care (*n* = 413) from 18 tertiary hospitals in Western, Eastern, Northern, and Southern China between May 2020 and June 2020. A structural equation modeling approach was then used to assess a hypothetical model wherein alexithymia both directly and indirectly affects burnout among emergency nurses via impacting the incidence of depression and perceived social support.

**Results:**

Results supported all driving hypotheses. Alexithymia was positive direct correlated with burnout (*β* = 0.35; *P* < 0.001) and depression (*β* = 0.50; *P* < 0.001), and exhibited a negative direct effect on social support (*β* = − 0.14; *P* = 0.041). Depression was associated with burnout, both directly (*β* = 0.24; *P* < 0.001) and indirectly (*β* = 0.15; *P* < 0.001) through its relationship with social support. Alexithymia was the factor most strongly associated with burnout, and it was able to affect burnout indirectly through depression and social support.

**Conclusions:**

We found that among emergency nurses in China, alexithymia was correlated with burnout, depression, and social support. Alexithymia was the factor most strongly associated with burnout. These data suggest that providing better social support and alleviating alexithymia may decrease rates of burnout among emergency nurses.

## Background

Alexithymia [1] is a personality trait associated with difficulties in affect regulation and describing one’s emotions that is linked to deficits in the cognitive–experiential emotional responses [2]. Alexithymic individuals often exhibit externally-oriented thinking, an inability to readily identify or describe subjective feelings, restricted imaginative processes, and may be unable to differentiate between feelings and bodily sensations of emotional arousal [1, 2]. Alexithymic individuals are thought to account for 7–10% of the general population [3–5], although some studies have found rates of alexithymia to be particularly high among nurses [6, 7]. Indeed, one study of 496 nurses in China found that 26% presented with alexithymia [8].

There is a positive correlation between alexithymia and the incidence of depression [9], and it is also a risk factor linked to the incidence of both physical and mental health conditions [2, 10]. Many studies have posited that alexithymia and depression may overlap or co-exist within individuals [11–13]. Tselebis and Kojima determined that the beneficial impact of familial and social support on symptoms of depression was reduced in individuals with alexithymic characteristics [14, 15]. Several prior analyses have also found that pronounced alexithymia was related to decreased social support and fewer close relationships [14, 16, 17]. A positive association between depression and alexithymia and a negative relationship between perceived familial support and alexithymia have also been reported among nurses [7].

Burnout was first conceptualized by Freudenberger in 1974, in light of observations that volunteers working with aid organizations exhibited symptoms of excessive stress after just a month of initial enthusiasm [18] Maslach and Jackson later expanded on this concept to define burnout as a series of symptoms including emotional exhaustion, depersonalization, and limited personal accomplishment linked to prolonged stress and job dissatisfaction [19, 20]. Rates of burnout are particularly high in careers that involve interactions with other people. Healthcare workers must reliably communicate with and provide care to patients, interact with the relatives of patients, and effectively work together with their coworkers in a team-based setting [21]. These complex and demanding factors are associated with high burnout rates, with one recent meta-analysis having reported a burnout rate of 51.98% among nurses [22]. Burnout rates were reported to be highest among nurses working in emergency departments [23–25].

Social support is a term referring to actual or perceived external resources available to an individual from their friends, family, partners, and coworkers [26]. A negative cross-correlation has been reported between social support and burnout among nurses [27], and social support is also a key factor that protects against depression both by providing positive social relationships and by indirectly buffering individuals against stress [28, 29]. Rates of depression among nurses are higher than those in the general population and are positively associated with burnout incidence [30–32].

In the context of a Job Demands-Resources (JD-R) model, burnout is a consequence of sustained high workplace demands for which personal and professional resources are insufficient to compensate. The resultant lack of balance can consume the energy of workers, resulting in mental exhaustion, adverse health effects, and negative effects for their employer as a consequence of this burnout [33]. This JD-R model incorporates personal resources as a relevant parameter, and as such these resources have been evaluated as mediators of the relationship between job characteristics and employee well-being [34]. These personal resources may explain why rates of burnout are variable even among employees in an identical working environment, with emotional skills functioning to modulate the ability of a given individual to avoid or manage burnout [35]. Alexithymia has also been consistently linked with higher burnout scores in the general population [7, 36], and the same has been found to be true among nurses [7, 37].

Past work has found that personal factors serve as key mediators of burnout incidence, potentially moderating or alleviating the challenges associated with integration into a constantly changing social setting [38–40]. Alexithymia, social support, and depression are all particularly relevant factors in this context given that they reflect the relationship between an individual and their contextual environment. Prior evidence suggests that all three of these variables are associated with burnout, but little is known regarding their comparative importance. There have also been few studies to date assessing the association between alexithymia and burnout among nurses in China.

As such, the present study was designed to analyze a population of emergency nurses in China in order to test the following three hypotheses: 1) that alexithymia is positively associated with burnout and depression and negatively associated with social support; 2) that depression is associated with burnout, both directly and indirectly through its relationship with social support; and 3) that social support and depression had mediating effects on the association between alexithymia and burnout.

## Methods

### Study design and participants

This was a descriptive cross-sectional survey of nurses from 18 tertiary hospitals in Western, Eastern, Northern, and Southern China (from the cities of Lanzhou, Dunhuang, Hangzhou, Qingdao, Nanning, Kunming, Beijing, Tianjin, and Dalian) conducted between May 2020 and June 2020. A convenience sampling method was used to enroll emergency nurses at these hospitals who were certified registered nurses with a minimum of 1 year of clinical nursing experience that agreed to participate in this study. Nurses not directly involved in patient care such as nurse managers, as well as nurses on rotation, vacation, or in training were excluded from this study. In total, 450 questionnaires were completed. Of the 450 received questionnaires, 37 were excluded from further analysis due to missing or invalid data, while the remaining 413(91.8%) were retained and used to estimate the model. The requirement for sample size for structural equation modeling (SEM) used in this study is that the ratio of the sample size to parameters should be no less than 10:1 [41]. Hence the sample size was acceptable to test a model with 29 free parameters, for which at least 290 cases were needed.

### Data collection

Nursing department directors at studied hospitals were contacted by an investigator associated with this study who explained the purpose of this research. After verbal consent had been provided to conduct data collection, questionnaires and informed consent documents were electronically distributed to each nursing department, and were printed for distribution among nurses at these hospitals. All nurses were informed of proper questionnaire distribution protocols. Completed questionnaires were returned by mail.

### Measures

#### Alexithymia assessment

The Chinese version of the 20-item Toronto Alexithymia Scale (TAS-20) [42], which has been utilized in prior studies of Chinese nurses [43], was used to assess alexithymia in the present study cohort. This scale is composed of seven items which measure difficulties associated with the identification of feelings (DIF subscale), five items that measure difficulties in describing feelings (DDF subscale), and eight items that assess externally oriented thinking (EOT subscale). A 5-point Likert scale ranging from 1 (‘strongly disagree’) to 5 (‘strongly agree’) was used to score all items, with total possible TAS scores ranging from 20 to 100. Higher scores were considered to be indicative of higher levels of alexithymia. The Chinese version of this scale exhibited good internal consistency for the total score, the DIF factors, DDF factors and EOT factors (Cronbach’s alpha of 0.83, 0.78, 0.61 and 0.55, respectively.)

#### Burnout measurement

The Chinese version of the Maslach Burnout Inventory - General Survey, which was translated and revised by Li et al. and is the most common tool used for studies of empirical burnout [44], was employed to assess burnout among emergency nurses in our study. The Chinese version of this survey has previously been leveraged to measure burnout among Chinese nurses [45], and is composed of 5 items measuring emotional exhaustion (EE), 4 items measuring depersonalization (DE), and 6 items measuring personal accomplishment (PA). Individual items were rated using a 7-point Likert scale (0 – never, 6 – daily), with higher EE and DE subscale scores and lower PA subscale scores being linked to higher levels of burnout. In the present study, all three subscales exhibited good internal consistency with Cronbach’s alpha values of 0.88, 0.83, and 0.82, respectively.

#### Depression assessment

The revised 21-item Beck Depression Inventory (BDI-II) [46] was used to assess depressive characteristics in study participants. All items were scored invidiously from 0 to 3 and then summed, yielding total BDI-II scores ranging from 0 to 63, with higher scores corresponding to more severe depressive symptoms according to the following cutoff criteria: minimal (0–13), mild (14–19), moderate (20–28), and severe (29–63). The Cronbach α was 0.92 of this study.

#### Social support measure

The Social Support Rating Scale (SSRS) [47], which is commonly used to assess Chinese populations, was used to assess social support in the present study. This scale consisted of 10 items measuring subjective support (SS, four items), objective support (OS, three items), and support availability (SA, three items). Item scores were summed together, with higher scores corresponding to greater social support. The Cronbach’s alpha for this scale in the present study was 0.91.

### Data analysis

SPSS v.25 (IBM, USA) was used for all statistical analyses in the present study. Relationships between the three dimensions of alexithymia, depression, the three dimensions of social support, and the three dimensions of burnout, as well as their multicollinearity (> 0.70), were analyzed using Pearson’s correlation coefficients [48]. No multicollinearity was detected in the present study.

Structural equation modeling (SEM) was conducted as a means of exploring associations among studied variables in the framework of the hypothesized model using Amos 23.0 (SPSS Inc., Chicago, IL, USA) with a maximum likelihood parameter estimation approach. SEM approaches define latent variables based on one or more observed variables, testing in a hypothetical and structured manner in order to confirm relationships among involved variables.

The Skewness and Kurtosis test was first employed to test the normality of study variables, with skewness values of − 0.91 to 1.09 and kurtosis values ranging from − 1.07 to 1.4 being indicative of univariate normality [48]. The Mardia’s coefficients for multivariate kurtosis in the present model was > 5, consistent with multivariate non-normality in the data. As such, a Bollen-Stine bootstrap p procedure was utilized to adjust model fit and parameter estimates so as to account for a lack of multivariate normality [49].

Multiple goodness-of-fit indices were employed when assessing model fit, including (1) χ^2^, (2) Comparative-of-Fit Index (CFI > 0.9), (3) χ^2^/df < 3, (4) Goodness-of-Fit Index (GFI > 0.9), (5) Tucker-Lewis Index (TLI > 0.9), and (6) the root mean square error of approximation (RMSEA < 0.06) values (BM, 2000).

The testing of structural models for this study relied upon both testing overall model fit and assessing the individual relationships between latent constructs. A two-tailed *P* < 0.05 was considered to be indicative of statistically significant effects for all analyses, and non-significant paths were excluded with the model being modified accordingly. To explore the indirect effects of the dependent variable through mediators, a bootstrap resampling method with 5000 bootstrap samples and 95% bias-corrected confidence intervals (*CIs*) around the standardized estimates of total, direct, and indirect effects was employed [50, 51].

### Results

#### Participant characteristics

The overall characteristics of participants in the present study are compiled in Table 1. These participants had a mean age of 31.06 ± 5.70 years (range: 20–55). Most study participants were married, female, and held a bachelor’s degree in nursing, with the mean work experience in the emergency department being 8.81 ± 5.92 years for this study population. The most common professional title among study participants was ‘senior nurse’ (74.3%), with 85.0% of total participants working as a contract nurse.

**Table 1:** Participants’ Characteristics (*N* = 413)

Characteristic	N(%)	
**Age, years (mean ± SD)**	31.06 ± 5.70	(range = 20–55)
**Sex**		
**Woman**	369	89.3
**Man**	44	10.7
**Marital status**		
**Married**	298	72.7
**Unmarried**	115	27.8
**Education level**		
**Associate degree or less**	96	23.2
**Bachelor’s or above**	317	76.8
**Work experience as a nurse, years (mean ± SD)**	8.81 ± 5.92	(range = 1–33)
**Professional title**		
**Nurse**	95	23.0
**Senior nurse**	307	74.3
**Nurse supervisor or above**	11	2.7
**Form of employment**		
**Contract nurse**	351	85.0
**Staff nurse**	62	15.0

#### Relationships between alexithymia, social support, depression, and burnout

Pearson’s correlation coefficient values corresponding to relationships between analyzed variables are shown in Table 2. Overall, these results revealed that all three dimensions of alexithymia were moderately positively correlated with all three dimensions of burnout and depression. All social support dimensions exhibited weak negative correlations with alexithymia, weak-to-moderate negative correlations with burnout, and negative correlations with depression. Of the three dimensions of burnout, both emotional exhaustion and depersonalization were highly correlated with depression, whereas only a weak correlation was observed between depression and personal accomplishment. Overall, these results revealed that all three dimensions of alexithymia were moderately positively correlated with all three dimensions of burnout and depression (*r* = 0.22–0.49, *p* < 0.001), and weakly negatively related to three dimensions of social support (*r* = − 0.14-0.31, *p* < 0.001).

**Table 2:** Pearson correlation coefficients among variables (*N =* 413)

Variables	M	SD	Range	1	2	3	4	5	6	7	8	9	10	11	12	13
**DIF(1)**	18.13	5.02	7–33	1												
**DDF(2)**	13.27	2.79	5–21	.811**	1											
**EOT(3)**	21.86	2.99	10–30	.471**	.445**	1										
**Alexithymia score (4)**	53.26	9.29	24–76	.936**	.882**	.710**	1									
**Emotional exhaustion (5)**	12.76	6.74	0–30	.451**	.351**	.216**	.419**	1								
**Depersonalization (6)**	7.13	5.35	0–24	.465**	.397**	.300**	.467**	.742**	1							
**Personal accomplishment (7)**	13.13	9.15	0–36	.364**	.322**	.328**	.399**	.105*	.255**	1						
**Burnout score(8)**	33.02	15.79	0–74	.561**	.471**	.384**	.569**	.740**	.804**	.711**	1					
**Subjective support (9)**	23.41	4.84	11–32	−.240**	−.199**	−.136**	−.233**	−.336**	−.379**	−.246**	−.415**	1				
**Objective support (10)**	9.57	3.32	1–18	−.153**	−.164**	−.156**	−.182**	−.191**	−.235**	−.152**	−.249**	.436**	1			
**Support availability (11)**	7.74	1.87	2–12	−.246**	−.306**	−.138**	−.269**	−.261**	−.332**	−.214**	−.348**	.310**	.335**	1		
**Social support score(12)**	40.72	7.85	19–61	−.271**	−.265**	−.183**	−.285**	−.350**	−.412**	−.267**	−.444**	.874**	.771**	.570**	1	
**Depression score(13)**	11.69	10.45	0–63	.486**	.386**	.309**	.478**	.501**	.506**	.204**	.504**	−.353**	−.319**	−.300**	−.423**	1

*P < 0.05, ** *P* < 0.001

*DIF* Difficulties with identifying feelings, *DDF* Difficulty of describing feelings, *EOT* Externally oriented thinking

#### Structural equation modeling

After initial structural equation modeling of actual parameter values, goodness-of-fit index values for the resultant model included χ^2^ = 135.51, χ^2^/df = 4.52, GFI = 0.94, TLI = 0.90, CFI = 0.93, and RMSEA = 0.09, consistent with a failure of these fitness indices to meet overall standards. Pull related error associated with emotional exhaustion and personal accomplishment dimensions in the burnout scale by modification index and the fitness indices for the modified model improved to χ2 = 102.03, χ2/df = 3.52, GFI = 0.96, TLI = 0.93, CFI = 0.95, and RMSEA = 0.08. However, the overall fit of this modified structural equation model was still sub-optimal. Given the detection of multivariate non-normality in the data, the Bollen-Stine bootstrap p procedure was then employed to adjust model fit, yielding index values of χ2 = 31.42, χ2/df = 1.08, GFI = 0.98, TLI = 1.00, CFI = 1.00, and RMSEA = 0.01. This model was thus sufficiently consistent with analyzed data to pass the verification test.

**Fig. 1:**
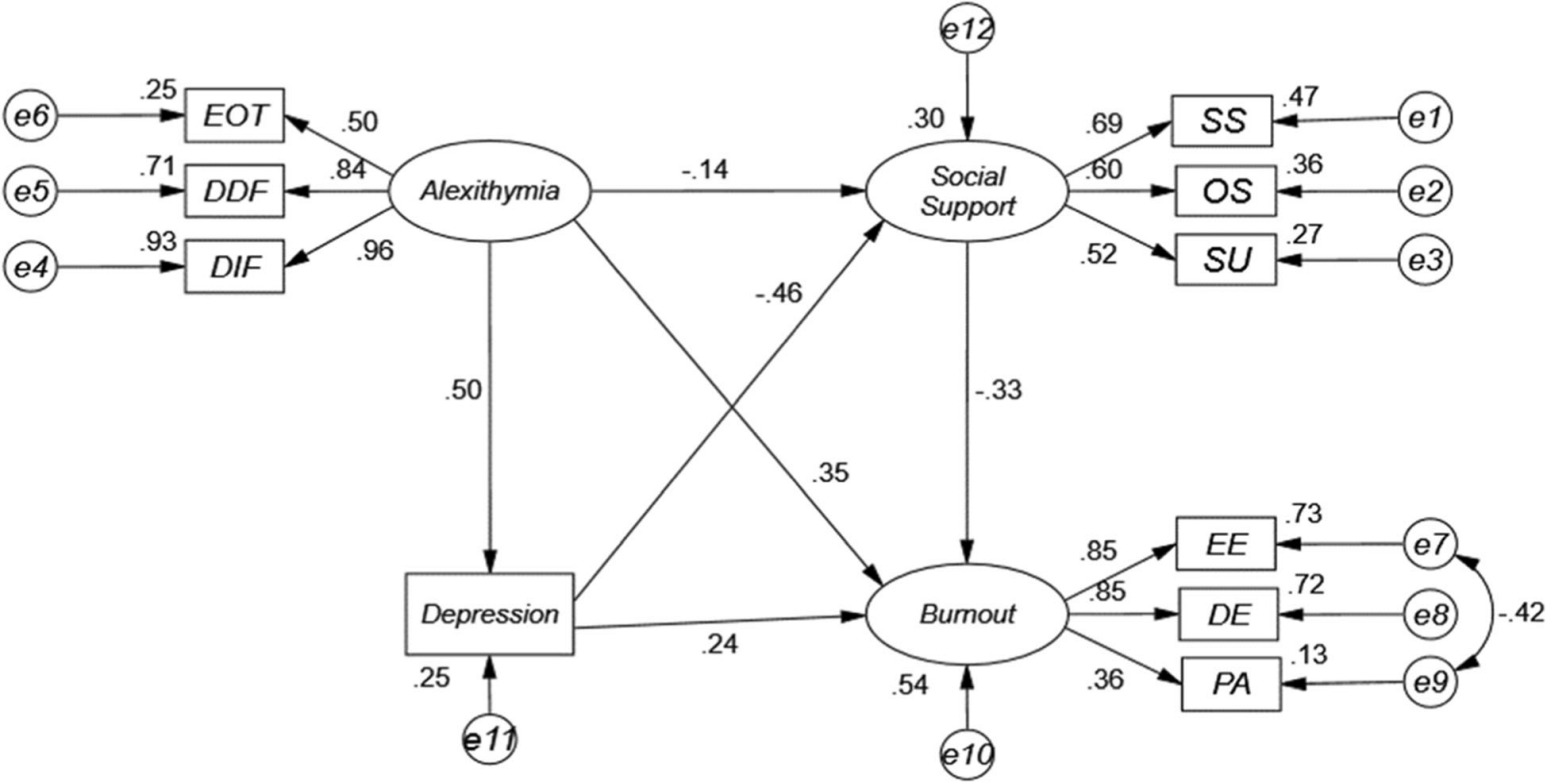
Estimated standardized direct effects for the final model. Ellipses represent latent factors. Squares represent measured variables (the scale or scale dimension scores). Arrows connecting circles and rectangles in one direction show a hypothesized direct relationship between two variables. Curved lines with an arrow in both directions demonstrate a bi-directional relationship (covariance). Circles with the letter “e” written in them represent the associated error. EOT, Externally oriented thinking; DDF, Difficulty of describing feelings; DIF, Difficulties with identifying feelings; SS, Subjective Support; OS, Objective Support; SA, Support Availability; EE, Emotional exhaustion; DE, Depersonalization; PA, Personal accomplishment

Direct path coefficients for this model are shown in Table 3 and Fig. 1, while bootstrap indirect effects are shown in Table 4. Specific outcomes associated with our three hypotheses are shown below:

**Table 3:** Regression weights among structural parameters

			Unstandardized direct effects	Standardized direct effects	S.E.	C.R.	***P***
**Depression←alexithymia**			1.079	0.500	0.102	10.603	< 0.001
**Social support←alexithymia**			−0.094	−0.137	0.046	−2.044	0.041
**Social support←depression**			−0.147	− 0.464	0.022	−6.729	< 0.001
**Burnout←social support**			−0.569	−0.328	0.124	−4.598	< 0.001
**Burnout←alexithymia**			0.414	0.349	0.062	6.633	< 0.001
**Burnout←depression**			0.132	0.240	0.032	4.192	< 0.001
**DIF←alexithymia**			1	0.964			
**DDF←alexithymia**			0.485	0.840	0.024	20.071	< 0.001
**EOT←alexithymia**			0.308	0.499	0.029	10.595	< 0.001
**Emotional exhaustion←burnout**			1	0.853			
**Depersonalization←burnout**			0.791	0.850	0.047	16.978	< 0.001
**Personal accomplishment←burnout**			0.574	0.361	0.092	6.204	< 0.001
**Subjective support←social support**			1	0.687			
**Objective support←social support**			0.598	0.599	0.071	8.395	< 0.001
**Support availability←social support**			0.293	0.522	0.038	7.746	< 0.001

Unstandardized direct effects come directly out of the estimation procedure. Due to the metric differences of the instruments, in this case, standardized direct effects should be preferred to indicate the strength of the associations (magnitude between −1 and + 1). Higher absolute values indicate a stronger (positive or negative) association. An absolute C.R. > 1.96 reflects that path coefficients are significant at the 0.05 level

*DIF* Difficulties with identifying feelings, *DDF* Difficulty of describing feelings, *EOT* Externally oriented thinking

**Table 4:** Bootstrap results for indirect effects among structural parameters

	Point Estimate	Product of Coefficients		Bootstrapping			
				Bias-corrected 95% CI		Percentile 95% CI	
		SE	Z	Lower	Upper	Lower	Upper
**Alexithymia→depression→burnout**	0.12	0.039	3.077	0.071	0.224	0.066	0.219
**Alexithymia→social support→burnout**	0.046	0.031	1.484	0.002	0.128	−0.002	0.123
**Alexithymia→depression→social support→burnout**	0.076	0.025	3.040	0.049	0.152	0.048	0.149
**Depression→social support→burnout**	0.15	0.022	6.818	0.046	0.136	0.046	0.135

5000 bootstrap samples

Hypothesis 1 - Alexithymia was associated with burnout, depression, and social support among Chinese emergency nurses.

We found that there was a significant positive direct correlation between alexithymia and burnout (β = 0.35; *P* < 0.001) as well as depression (β = 0.50; *P <* 0.001). Alexithymia also exhibited a negative direct effect of − 0.14 (*P* = 0.041) on social support (Table 3 and Fig. 1), and was able to explain 25% of the variance in the impact of depression (*R*^*2*^ = 0.25) (Fig. 1).

Hypothesis 2 – Depression was associated with burnout, both directly and indirectly through its relationship with social support.

We found that depression had a total effect of 0.39 on burnout, including a direct effect of 0.24 (*P* < 0.001) and an indirect effect of 0.15 (*P <* 0.001) through social support (Tables 3, 4 and Fig. 1). Together, alexithymia and depression explained 30% of the variance of social support (*R*^*2*^ = 0.30) (Fig. 1).

Hypothesis 3 - Social support and depression had mediating effects on the association between alexithymia and burnout.

Alexithymia had a total effect of 0.59 on burnout, of which 0.24 (*P <* 0.001) was an indirect effect through social support and depression, indicating a mediating influence on this relationship (Tables 3, 4 and Fig. 1). With respect to the mediating effects of depression, the indirect effect of burnout through depression was 0.12, and the total effect on burnout was 0.59. The mediating effect (indirect effect/total effect) of depression was 20.34%. With respect to the mediating role of social support, the indirect effect of burnout through social support was 0.046 (*P* > 0.05), with no significant mediating effect of social support in this context. With respect to the mediating roles of depression and social support, the indirect effect of burnout through depression and social support was 0.08, and the mediating effect of depression and social support was 12.89%. Together, alexithymia, social support, and depression explained 54% of the variance of burnout (*R*^*2*^ = 0.54) (Fig. 1).

## Discussion

This study is among the first to provide support for a comprehensive model of the relationship between alexithymia, depression, and social support as mediators of burnout among Chinese emergency nurses. The results of our multi-center survey revealed that burnout incidence was positively correlated with alexithymia and depression, and was mitigated by social support, with similar weights being associated with these different relationships. We also found that depression and social support mediated and mitigated the relationship between alexithymia and burnout. In this studym, we found that alexithymia was the factor most strongly associated with burnout, and we also found that it was able to affect burnout indirectly through depression and social support. It is noteworthy that the study found that personal accomplishment of burnout is positively related to emotional exhaustion, depersonalization, and depression, and negatively correlated to positive outcomes. A reasonable explanation may be that in the revised Chinese version of MBI-GS, PA (personal accomplishment) stands for low personal accomplishment, which mainly evaluates the degree of reduced competence and accomplishment experienced by individuals at work.

Decreasing alexithymia represents a potentially viable approach to alleviating the incidence of burnout among emergency nurses, given that our model confirmed our hypothesis that alexithymia had both direct and indirect effects on burnout in this population. These data were also consistent with prior research pertaining to a correlation between alexithymia and burnout [7, 37]. Awareness of alexithymic traits is of particular importance in a healthcare setting, given that a lack of emotional awareness can make it extremely challenging to establish the functional relationships with patients that are required to conduct accurate medical examinations [52]. The ability to define one’s own emotions is also key to the appropriate management of stressful working environments, and this ability is likely to be limited in alexithymic individuals [52–54]. A few studies to date have identified effective approaches to ameliorating alexithymia, including group psychotherapy, artistic learning, expressive writing, and reading literary fiction [55–57]. The implementation of these or other approaches to reducing alexithymia in a hospital management context may lower rates of nurse burnout.

Depression was related to burnout among nurses both directly, as well as indirectly via social support. Depression was also a key mediator of the association between alexithymia and burnout. Overall, our results were consistent with our hypothesis and were also in line with prior analyses of the relationship between burnout and depression [30–33]. We found that individuals with depressive characteristics were more vulnerable to burnout, potentially due to their inability to derive satisfaction from their careers [58]. The mediating role of depression in our analysis suggests that nursing managers should seek to decrease the impact of depression on burnout among emergency nurses by ameliorating alexithymia where possible.

Our data revealed that social support functioned as a remote mediator of the relationship between alexithymia and burnout through depression. In line with our hypothesis and other prior reports [7, 27, 59, 60], we determined that greater access to social support was protective against burnout, and was also sufficient to regulate the association between alexithymia/depression and burnout. The relative weights of the direct effects of social support and alexithymia on burnout were also similar in this analysis, underscoring the importance of social support and indicating that individuals responsible for managing nurses should work to improve employee access to this important resource in order to alleviate burnout rates among emergency nurses. Among healthcare professionals, peer support has been found to be a particularly valuable type of social support [61], enabling nurses to communicate regarding their work-related stressors and to prevent or manage burnout. However, we did not observe any direct mediating effect of social support on alexithymia and burnout among nurses in the present study. This may suggest that individuals with alexithymic characteristics are less able to benefit from available social support, although further analyses will be necessary to validate this finding.

There are multiple limitations to this analysis that warrant consideration when interpreting our results. First, while nurses were recruited from 18 hospitals, this analysis was nonetheless limited to a population of emergency nurses. These results may therefore not be generalizable to nurses working in other clinical settings. Second, we only assessed unidirectional relationships among study variables, and we were thus not able to generate causal inferences through this cross-sectional study design. Future prospective longitudinal studies will be required to validate and expand upon our findings. Third, a self-reporting approach was employed to evaluate study participants, potentially biasing study results. Fourth, other factors not considered in this study may have influenced the variables analyzed herein, as confounding variables were not accounted for in this study.

### Recommendations for nursing practice and policy

Despite these limitations, these data have important clinical implications and theoretical implications for the formulation and implementation of subsequent interventions. In the future clinical work, administrators and nursing managers should consider the alleviation of alexithymia as a potentially viable means of reducing the incidence of burnout among emergency nurses, particularly when social determinants of burnout are not amenable to change. In addition, perhaps when interviewing new nurses before they start working in an emergency department the administrators should screen the nurses for alexithymia as a preventative measure, which can help nurses effectively cope with burnout, and ultimately reduce the high rates of burnout.

## Conclusions

In summary, we found that alexithymia was positive direct correlation with burnout and depression, and exhibited a negative direct impacts on social support. Depression was associated with burnout both directly and indirectly through effects on social support. Social support and depression played a mediating role in influencing the association between burnout and alexithymia. We propose that improving alexithymia and providing social support to these nurses may help to reduce the high rates of burnout observed among these professionals.

## Data Availability

The datasets used and/or analysed during the current study are available from the corresponding author on reasonable request.

## Abbreviations

EOT: Externally oriented thinking
DDF: Difficulty of describing feelings
DIF: Difficulties with identifying feelings
SS: Subjective Support
OS: Objective Support
SA: Support Availability
EE: Emotional exhaustion
DE: Depersonalization
PA: Personal accomplishment
